# Non-dimensional groups for electrospray modes of highly conductive and viscous nanoparticle suspensions

**DOI:** 10.1038/s41598-020-61323-5

**Published:** 2020-03-10

**Authors:** Eduardo Castillo-Orozco, Aravinda Kar, Ranganathan Kumar

**Affiliations:** 1 Escuela Superior Politecnica del Litoral, ESPOL, Facultad en Ingenieria Mecanica y Ciencias de la Produccion, Campus Gustavo Galindo, Km. 30.5 Via Perimetral, Guayaquil, P.O. Box 09-01-5863 Ecuador; 2 CREOL, The College of Optics and Photonics, University of Central Florida, 4000 Central Florida Blvd, Orlando, Florida 32816 USA; 3 Department of Mechanical and Aerospace Engineering, University of Central Florida, 4000 Central Florida Blvd, Orlando, Florida 32816 USA

**Keywords:** Fluid dynamics, Characterization and analytical techniques

## Abstract

Multiple modes of atomization in electrosprays are affected by viscosity, surface tension and electrical conductivity of the semiconductor nanosuspensions. While the effect of gravity is dominant in the dripping mode, the electric field degenerates the electrospray mechanism into a microdripping mode that can potentially allow the deposition of semiconductor nanodots on a substrate. Drop size and frequency of droplet formation are obtained as functions of non-dimensional parameters, which agree well with experimental data. The analysis shows that it is possible to produce the desired size and frequency of ejection of monodisperse droplets by manipulating the electrode voltage for any nanosuspension.

## Introduction

Patterning of semiconductor structures has a crucial application for fabricating electronic and photonic devices^1,2^. Nanoparticles offer the additional benefit of low melting temperature and better mechanical and optical properties compared to the bulk material. Electrosprays present a convenient method to carry these nanoparticles within droplets for deposition of semiconductor materials, e.g., copper-indium-diselenide for solar cells^3^ and zinc oxide for thin-film transistors^4^.

The physics of electrosprays has been discussed in two critical reviews^5,6^. The electric forces in microfluidic systems can cause various phenomena including transient, periodic, quasi-steady and steady flows. The electrospray mostly used operates in a steady cone-jet spray mode where micro- and nano-droplets are detached from the tip of a capillary tube due to electric force^7–10^, but in general, it can operate in numerous modes depending on the flow rate and electric potential^11–13^. These modes have been used in inkjet printing with sub-micrometer resolution and minimal clogging^14,15^.

Semiconductor nanoparticles can be suspended in water and electrosprayed on substrates for additive manufacturing of electronics. This was demonstrated in our recent study^16^ where low viscosity suspensions were investigated. In electrosprays, the droplets detach from the tip of a capillary tube due to the balance of the electrodynamic and hydrodynamic forces acting on the fluid. An electrostatic field between the capillary tube and an extraction electrode in the vicinity of the tube alters the mode of detaching the droplets, resulting in different spray characteristics such as dripping, microdripping, spindle, multispindle, and cone-jet spray mode^11,17–19^. Thus, the electro-capillary interaction provides an additional mechanism to generate different modes for the fluid dynamic response of the droplets.

The Taylor Cone jet is formed when high voltage is applied to the capillary tube and the liquid is polarized. The electric field around the liquid flowing downward from the capillary tube deforms the liquid meniscus into a Taylor cone from which the micron-sized droplets are emitted. The electrical conductivity and the liquid flow rate can affect the dropsize^9,20^.

The microdripping mode, which is a stable method to generate uniform droplets carrying semiconductor nanoparticles for deposition on substrates, follows the initial dripping mode that occurs at lower electric potential. The production of fine monodisperse droplets from a precursor suspension is of interest. These suspensions can have high viscosity and high density due to high particle concentration^21,22^. Hijano *et al*.^23^ found that in an electrospray of low viscosity fluid, the process depends on two non-dimensional parameters: normalized flow rate and electric Bond number which is a measure of the square of the voltage. The current paper deals with the inception of a viscous droplet in the dripping regime as well as the microdripping regime. Hence, the relevant non-dimensional parameters that will be discussed include electric capillary number (similar to electric Bond number used in^23^), viscous capillary number and Bond number.

In our early work^16^, low viscosity suspension of capillary number (*Ca*) of 0.1 or less were used. Therefore, the viscous effect is not significant to include *Ca* as a parameter, and drop size and frequency could be plotted as a simple function of electric capillary number (*Ca*_*e*_), Bond number (ratio of gravity to surface tension force) was not explicitly considered. In this paper, a more detailed theoretical analysis will be reported. This paper focuses on high viscosity suspensions of 0.03 < *Ca* < 0.4 with high electrical conductivity. For these suspensions, the viscous effects become important and affect the conical meniscus. Here, in addition to the interfacial, gravity and electric forces, viscous force will play a decisive role in the non-dimensional analysis. Experimental results for droplet diameter and drop frequency will be presented in terms of a unique combination of relevant non-dimensional parameters such as electric Capillary number, viscous Capillary number, and Bond number that are derived from a simple force balance. Correlation will be developed for non-dimensional radius and frequency of emission of droplets in terms of the non-dimensional group *Ca*_*e*_/[*Bo*(1 + *Ca*)]^0.5^.

## Results

The experiments were performed for a number of suspensions with a wide range of physical properties, voltages from 0 to 5000 V (Table 1). The presence of semiconductor nanoparticles inside the droplets, particularly the electrical conductivity of the nanomaterials, will influence the transition of the microdroplet modes. Experiments were carried out at low flow rates corresponding to Stokes flow to investigate the modes of nanosuspensions in laminar electrospray of droplets.

**Table 1:** Physical properties and relevant parameters of semiconductor nanosuspensions in DI water.

Semiconductor	*ρ*	*μ*	*σ*	*γ*	^(*c*)^*r*_0_	^(*e*)^*r*_0_	*Ca*	*Bo*	*Ca*_*e*_
nanosuspension	(g/ml)	(mPa.s)	(*μ*S/cm)	(mN/m)	(mm)	(mm)			
Si, 2 wt% in DI water	1.010	1.48	991.6	46.7	1.20	1.18	0.035	0.202	0–2.91
Si, 5 wt% in DI water	1.033	1.82	1391.6	48.2	1.21	1.19	0.031	0.204	0–2.90
Si, 10 wt% in DI water	1.060	2.72	1411.2	47.1	1.19	1.13	0.083	0.209	0–3.21
SiC, 2 wt% in DI water	1.051	1.56	484.0	47.7	1.19	1.19	0.048	0.203	0–2.61
SiC, 5 wt% in DI water	1.127	4.79	545.0	48.5	1.20	1.19	0.139	0.220	0–2.57
SiC, 10 wt% in DI water	1.193	12.05	582.0	59.9	1.19	1.23	0.279	0.151	0–2.69
ZnO, 10 wt% in DI water	1.103	1.46	741.0	55.1	1.25	1.29	0.041	0.190	0–3.08
ZnO, 30 wt% in DI water	1.438	2.09	1400.0	52.4	1.07	1.08	0.055	0.204	0–2.88
ZnO, 50 wt% in DI water	1.930	18.50	1782.0	54.3	1.05	1.07	0.405	0.254	0–2.92

^(c)^Drop radius in the absence of electric field, calculated using Eq. (6).

^(e)^Drop radius in the absence of electric field from experiments.

Electrospray in the dripping mode has negligible inertial and viscous effects^24,25^, while in the microdripping mode the viscosity affects the conical meniscus at the tip of the capillary tube from which the droplets are detached. Both modes are inherently quasi-static for a constant flow rate of the suspension into the capillary tube. In addition, a fluid of higher viscosity has the advantage of decreasing the jet diameter^26^. The droplet grows to a steady-state size and detaches from the tube, and this process repeats in a periodic manner to ensure the discharge of droplets at a certain frequency. The droplet radius observed in this study is between 1.3 mm–0.65 mm in the dripping mode and 74 *μ*m–150 *μ*m in the microdripping mode. The droplet formation rates are measured between 0.2–1.5 droplets per second in the dripping mode and 100–650 droplets per second in the microdripping mode, respectively.

**Figure 1:**
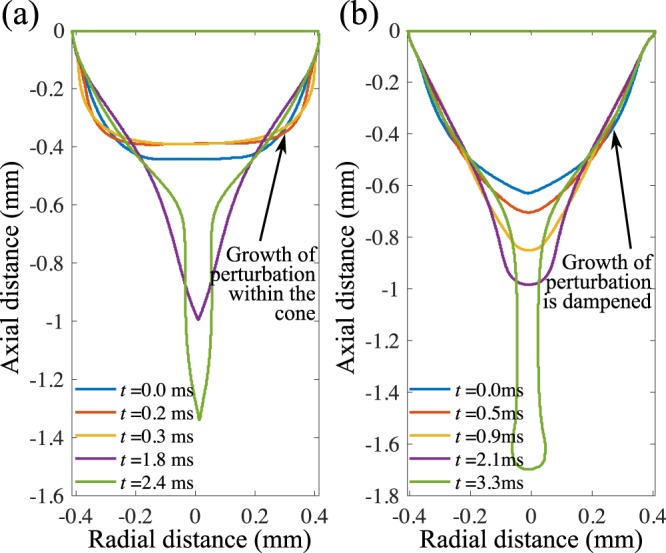
Comparison of the sequence of growth of the image-processed conical meniscus due to the action of an electric force. (**a**) Microdripping of low viscosity suspension (Si 10 wt% in DI water, *Ca* = 0.083, *ϕ* = 4000 V). (**b**) Microdripping of high viscosity suspension (SiC, 10 wt% in DI water, *Ca* = 0.279 *ϕ* = 5000 V).

In the dripping mode, the droplets are formed from a hemispherical or ellipsoidal meniscus, while the electric field-induced stretching of the fluid develops a conical meniscus called the Taylor cone. A neck develops between the meniscus and a pendant liquid filament at the end, which becomes thinner with time and eventually collapses releasing a droplet. This droplet ejection process is periodic and monodisperse. Figure 1 compares the growth of the conical meniscus due to the presence of electric force in the microdripping mode both in low viscous and high viscous suspension. Here, the conical meniscus and hence the ejected droplet are relatively small, as is the gravity force. For the high viscosity fluid, the electric force stretches the liquid to form the meniscus and the liquid filament, while the viscous friction prevents the elongation of the liquid filament, and the surface tension force first resists the formation of this pendant filament, but once the droplet is detached, the restoring force of the capillary effects turns the spindle-shaped liquid filament into a spherical droplet. The relative influence between the viscous and interfacial force is seen when the capillary number increases in the microdripping mode. In contrast, the velocity is lower in the dripping mode and the evolution of the ellipsoidal meniscus is weakly influenced by the lower capillary number. Thus, the effect of viscosity is minimal in the dripping mode, however, in the microdripping mode, the droplet size increases slightly with increasing viscosity and the apex of the meniscus stretches more in highly viscous suspensions (Fig. 1). Viscosity affects the shape of the cone by resisting its deformation and thus promotes a stable microdripping mode.

**Figure 2:**
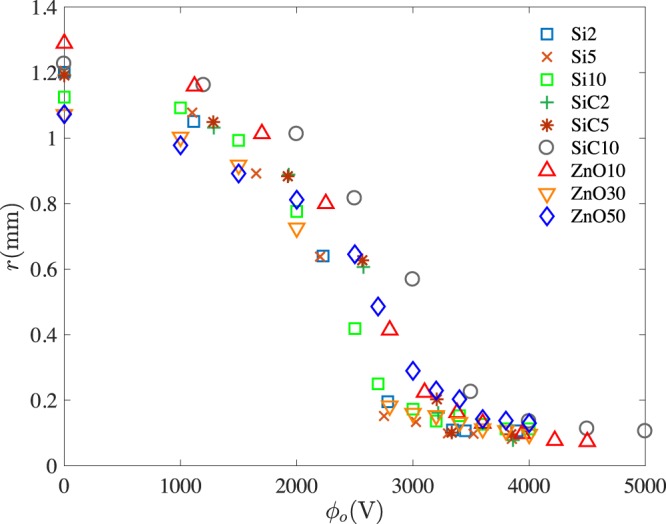
Radius of the emitted droplets vs. applied voltage in the dripping and microdripping modes for *Q* = 1.67 mm^3^/s.

In general, the capillary forces increase the neck radius to minimize the curvature of the liquid-air interface, while the gravity and electric force stretch the interface until they balance the viscous friction and capillary force. For all 9 suspensions (Fig. 2), the droplet diameter in the dripping mode decreases up to  ≈ 2000 V, but with a wide scatter. From this point to  ≈ 3000 V, depending on the suspension, the droplets are either in dripping mode or microdripping mode. This regime between the dripping and microdripping modes is characterized as mixed mode. Otherwise, for a given suspension and a set of physical properties, dripping mode smoothly transitions into microdripping mode where the radius can be as low as 0.1 times the original radius. The scatter in the data continues to be high for these suspensions in both modes.

**Figure 3:**
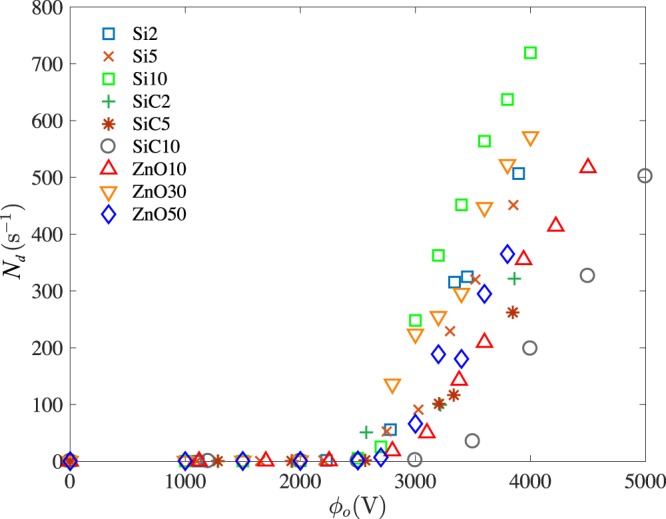
Frequency of droplet formation vs applied electric voltage for the same conditions as in Fig. 2.

The frequency of droplet formation increases with higher electric voltages (Fig. 3). This frequency is influenced by the formation of the conical meniscus, which occurs at different voltages for different suspensions, i.e., at 2500 V for Si, 10 wt% in DI water and 3500 V for SiC, 10 wt% in DI water. This is also the reason for different frequencies of droplet formation at a given voltage for different suspensions. For example, for the above two suspensions, 200 and 650 droplets per second are ejected from the capillary tube respectively at 4000 V. The frequency changes abruptly at the transition between dripping and microdripping mode. Fluids with high capillary number tend to have lower frequency at a given electric capillary number due to viscous effects. As in the case of droplet radius, the frequency data also displays a large scatter, which provides motivation to find non-dimensional parameters to minimize the scatter in both droplet radius and frequency.

### Nondimensional analysis

Scaling laws have been proposed by^7,23,27^ to predict the size and charge of the emitted droplets. In this work, since the scatter is high in the raw data, proportionality relations are needed, which can be useful to designers especially since microdripping occurs in a very narrow range of voltage for a wide range of fluids with different physical properties. In the steady cone-jet mode, the meniscus can be assumed to be in stable quasi-conical shape from where a thin jet is ejected, which then breaks up into droplets due to capillary and viscous forces.

Since both dripping and microdripping modes are inherently quasi-static for a constant flow rate, a simple steady-state force balance can be applied at the onset of detachment to obtain relevant parameters. These parameters can then be grouped to arrive at a single parameter which can be used to plot all the data for the 9 suspensions with different properties as given in Table 1. The intent is to develop non-dimensional parameters to plot the data with limited scatter and use these parameters in both dripping and microdripping regimes. Therefore, an assumption is made that the process of droplet detaching from the capillary tube repeats in a periodic manner to ensure the discharge of droplets at a certain frequency. The forces present on the droplet at the onset of detachment from the meniscus are: the hydrodynamic forces, i.e., viscous, and interfacial forces; the gravitational force and the electric force. The force balance is applied, which is valid for either the ellipsoidal and conical meniscus, i.e., dripping and microdripping mode. However, different conditions must be applied for each mode which may include some empiricism, thus, yielding different expressions that estimate normalized droplet radius as a function of non-dimensional parameters for each regime.

The electric force, *F*_*Electric*_, is estimated to be 2*πr*^2^*ε*_0_*ε*_*r*_*E*^2^ (see Appendix for details) where *r* is the radius of the droplet; *ε*_0_, *ε*_*r*_ and *E* are the electric permittivity of vacuum, characteristic relative permittivity of each aqueous suspension, and the externally applied electric field. The gravitational force is *F*_*Gravity*_ = 4*πr*^3^*ρg*/3, where *ρ* and *g* are the fluid density and gravitational acceleration. Although the electric and gravitational forces are initially pulling on the elongated filament, at the inception of the drop, the radius of the filament becoming a droplet has been used to estimate the two forces.

**Figure 4:**
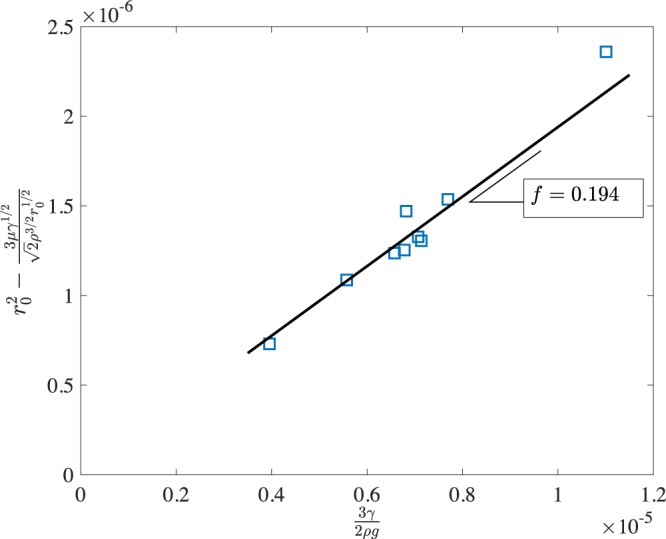
Experimental factor *f* from experimental data.

The electric and gravitational forces are resisted by the interfacial and viscous forces. The surface tension force restores the deformed droplet into a sphere. This interfacial force, *F*_*Interfacial*_ is estimated to be 2*πwγ*, where 2*πw* is the circumference of the meniscus in the neck area and *γ* is the surface tension. *w* = *f* ⋅ *r*, where *f* is Harkins correction factor for the range of droplet size in the current experiments, following^25,28^. The Harkins correction factor is obtained as 0.194 from Fig. 4, which allows the results to be obtained within 10% error.

The viscous force is estimated as follows. The viscous force is the shearing force at the neck of the hanging liquid mass that depends on the liquid viscosity, *μ*, the rate of shear strain, and the surface area produced by the arc length of liquid neck (2*πr*) and a characteristic viscous length, *l*. The shear rate (∂*u*/∂*y*) is scaled as (*u*_*c*_/*l*). Thus, the viscous shearing force is *F*_*Viscous*_ = *μu*_*c*_2*πr*^29^. At the onset of detachment from the meniscus, according to Hijano *et al*.^23^, the time can be scaled by the capillary time and the distance by the dropsize, *r*_0_, at *E* = 0. The capillary time is ${t}_{c} \sim {(\rho {r}_{0}^{3}/\gamma )}^{1/2}$ which yields a velocity scale, ${u}_{c} \sim {\left(\frac{\gamma }{\rho {r}_{0}}\right)}^{1/2}$. Note that the length scale used by Hijano *et al*.^23^ was the needle diameter. In this paper *u*_*c*_ is estimated by considering an energy balance between inertia and surface energy per unit area, which yields:

1 $${u}_{c}=\sqrt{2\gamma /{r}_{0}\rho }$$

where *r*_0_ is the radius of the largest droplet for *E* = 0.

A simple force balance for the droplet at the time of ejection,

2 $${F}_{Electric}+{F}_{Gravity}\approx {F}_{Interfacial}+{F}_{Viscous}$$

Equation (2) now becomes

3 $$2\pi {r}^{2}{\varepsilon }_{0}{\varepsilon }_{r}{E}^{2}+\frac{4}{3}\pi {r}^{3}\rho g=2\pi rf\gamma +2\pi r\mu {u}_{c}$$

Speranza and Ghadiri^25^ used an expression similar to Eq. (3) without the viscous force.

Solving the quadratic Eq. (3),

4 $$r=\frac{\sqrt{{({\varepsilon }_{0}{\varepsilon }_{r}{E}^{2})}^{2}+\frac{8}{3}\rho g(f\gamma +\mu {u}_{c})}-{\varepsilon }_{0}{\varepsilon }_{r}{E}^{2}}{\frac{4}{3}\rho g}$$

From Eq. (4), the radius *r*_0_ is obtained as

5 $${r}_{0}=\sqrt{\frac{3(f\gamma +\mu {u}_{c})}{2\rho g}}$$

Using this expression of *r*_0_, the following algebraic equation is obtained for determining *r*_0_ in terms of the physical properties of the droplets.

6 $${r}_{0}^{5/2}-\frac{3f\gamma }{2\rho g}{r}_{0}^{1/2}-\frac{3\mu {\gamma }^{1/2}}{\sqrt{2}{\rho }^{3/2}}=0$$

where the correction factor was empirically found to be *f* = 0.194 (Fig. 4). The values of *r*_0_ calculated using equation (6) and determined from experimental data are listed in Table 1.

Now the normalized drop size, (r^*^ = *r*/*r*_0_) is obtained from Eq. (6) which becomes

7 $$r^{\ast}=\sqrt{1+\frac{1}{4}\frac{{({\varepsilon }_{0}{\varepsilon }_{r}{E}^{2})}^{2}}{\frac{2}{3}\rho g(f\gamma +\mu {u}_{c})}}-\frac{1}{2}\frac{{\varepsilon }_{0}{\varepsilon }_{r}{E}^{2}}{\sqrt{\frac{2}{3}\rho g(f\gamma +\mu {u}_{c})}}$$

Rewriting Eq. (7) in terms of non-dimensional parameters, electric Capillary number (ratio of electric to surface tension forces): *Ca*_*e*_ = *E*^2^*e*_0_*e*_*r*_*R*_0_/*γ*; nominal Bond number (ratio of the gravity to surface tension forces): $Bo=2{r}_{0}^{2}\rho g$/3*γ*, and modified Capillary number (ratio of viscous to surface tension forces), *Ca* = *μu*_*c*_/*fγ*,

8 $$r^{\ast}=\sqrt{1+\frac{{r}_{0}^{2}}{4{R}_{0}^{2}}\frac{C{a}_{e}^{2}}{Bo(1+Ca)}}-\frac{{r}_{0}}{2{R}_{0}}\frac{C{a}_{e}}{\sqrt{Bo(1+Ca)}}$$

The electric force and the surface tension force are balanced when *Ca*_*e*_ = 1 and around this value, the dripping mode transitions into microdripping mode.

For the dripping mode, Eq. (8) can be rewritten as follows after binomial expansion when ${r}_{0}^{2}C{a}_{e}^{2}$/$4{R}_{0}^{2}Bo(1+Ca) < 1$:

9 $$r^{\ast}=1-\frac{{r}_{0}}{2{R}_{0}}\left(1-\frac{{r}_{0}}{4{R}_{0}}\frac{C{a}_{e}}{\sqrt{Bo(1+Ca)}}+..\right)\frac{C{a}_{e}}{\sqrt{Bo(1+Ca)}}$$

For the microdripping mode, Eq. (8) is written as

10 $$r^{\ast}=\frac{{r}_{0}}{2{R}_{0}}\frac{C{a}_{e}}{\sqrt{Bo(1+Ca)}}\sqrt{1+\frac{4{R}_{0}^{2}}{{r}_{0}^{2}}\frac{Bo(1+Ca)}{C{a}_{e}^{2}}}-\frac{{r}_{0}}{2{R}_{0}}\frac{C{a}_{e}}{\sqrt{Bo(1+Ca)}}$$

which can be simplified as follows by binomial expansion when $4{R}_{0}^{2}Bo(1+Ca)$/${r}_{0}^{2}C{a}_{e}^{2} < 1$:

11 $$r^{\ast}=\frac{{R}_{0}}{{r}_{0}}\left(1-\frac{{R}_{0}^{2}}{{r}_{0}^{2}}\frac{Bo(1+Ca)}{C{a}_{e}^{2}}+..\right)\frac{\sqrt{Bo(1+Ca)}}{C{a}_{e}}$$

Thus, defining the coefficients *δ*_1_ and *δ*_2_ as $({r}_{0}/2{R}_{0})(1-{r}_{0}C{a}_{e}/4{R}_{0}\sqrt{Bo(1+Ca)}+...)$ and $({R}_{0}/{r}_{0})(1-{R}_{0}^{2}Bo(1+Ca)/4{r}_{0}^{2}C{a}_{e}^{2}+...)$ respectively, the normalized drop size can be predicted as shown in Eq. (12) and (13), where these coefficients are to be determined empirically.

12 $$r^{\ast}=1-{\delta }_{1}\frac{C{a}_{e}}{\sqrt{Bo(1+Ca)}};{\rm{Dripping}}$$

13 $$r^{\ast}={\delta }_{2}{\left(\frac{C{a}_{e}}{\sqrt{Bo(1+Ca)}}\right)}^{-1};{\rm{Microdripping}}$$

Next, the number of emitted droplets from the meniscus is estimated to be *N*_*d*_ = 3*Q*/4*πr*^3^ and is normalized as ${N}_{d}^{\ast}={({N}_{d}{r}_{0}^{3}/Q)}^{1/3}$. Thus, ${N}_{d}^{\ast}$ for dripping and microdripping mode is determined as follows,

14 $${N}_{d}^{\ast}={\left(\frac{3}{4\pi }\right)}^{1/3}{\left(1-\frac{{\delta }_{1}C{a}_{e}}{\sqrt{Bo(1+Ca)}}\right)}^{-1};{\rm{Dripping}}$$

15 $${N}_{d}^{\ast}={\left(\frac{3}{4\pi }\right)}^{1/3}\frac{C{a}_{e}}{{\delta }_{2}\ \sqrt{Bo(1+Ca)}};{\rm{Microdripping}}$$

Note that the force balance contains four different forces where the gravity force is given as an exact term, and a correction factor *f* is introduced for the interfacial force. However, the other two forces, i.e., electric and viscous, represent estimated expressions. To account for these approximations, *δ*_1_ and *δ*_2_ are introduced as correction factors in Eq. (12) through (15), and are found based on the experimental data for nine suspensions of varying viscosities and electrical conductivities over a wider range, tested by increasing the voltage. Due to the simplicity of the force balance, these expressions should be viewed as correlations based on a unique combination of non-dimensional parameters, *Ca*_*e*_/$\sqrt{Bo(1+Ca)}$. For low viscosity suspensions and constant *Bo*, the normalized drop radius and frequency are functions of electric capillary number, called as electric Bond number by Hijano *et al*.^23^.

In the microdripping mode, an increase in voltage increases the electric field and causes a change in the shape of the meniscus and ejects smaller droplets at a higher frequency of emission. Therefore, the normalized radius is an inverse function of *Ca*_*e*_. With increase in viscosity, i.e., capillary number, *Ca*, the normalized radius increases as (1 + *Ca*)^0.5^. For low viscosity suspensions, (1 + *Ca*)^0.5^ ~ 1. The normalized frequency has the opposite effect in that it increases with the electric field, i.e., *Ca*_*e*_ and is inversely proportional to (1 + *Ca*)^0.5^. Since the suspensions have been tested for a wide ranging viscosity with an order of magnitude increment, it is important that the frequency has been derived to be inversely proportional to (1 + *Ca*)^0.5^ which again reduced to  ~ 1 for low viscosity suspensions.

### Data analysis

**Figure 5:**
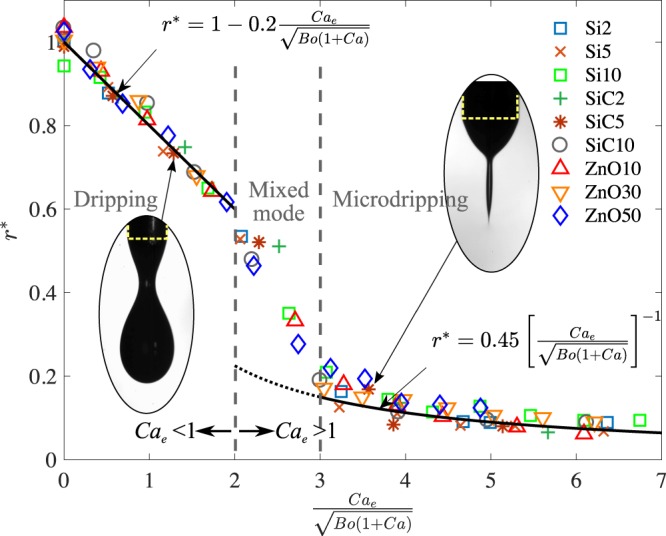
Normalized drop radius vs the ratio between the electric capillary number and the Bond and capillary number. Symbols represent the experimental data for the same suspensions given in Fig. 2, while the solid lines are the Eqs. (12) and (13) for dripping and microdripping mode, respectively. *δ*_1_ = 0.2  (12) and *δ*_2_ = 0.45  (13).

**Figure 6:**
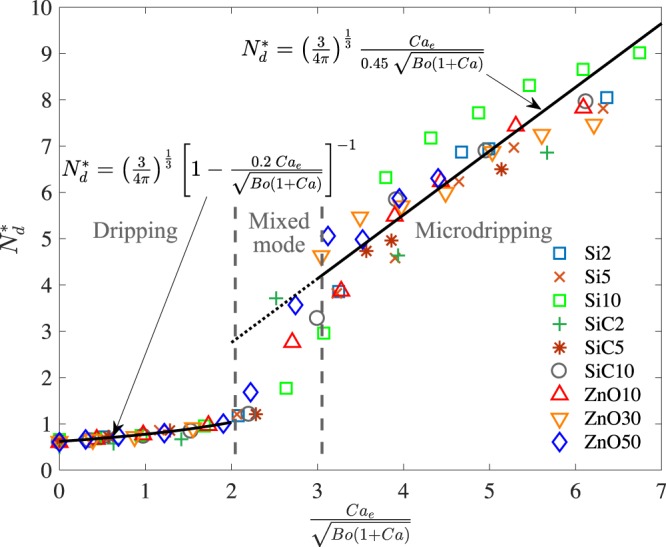
Non-dimensional frequency of droplet formation vs the ratio between the electric capillary number and the Bond and capillary number. Symbols represent the experimental data and the solid lines are Eqs. (14) and (15) for dripping and microdripping mode.

The analysis provided in equations (12) through (15) fit the data well (Fig. 5) showing that r^*^ is a linearly decreasing function of *Ca*_*e*_/$\sqrt{Bo(1+Ca)}$ in the dripping mode in the range *Ca*_*e*_/$\sqrt{Bo(1+Ca)} < 2$, and a hyperbolic function for 3 < *Ca*_*e*_/$\sqrt{Bo(1+Ca)} < 7$. This suggests that this group of parameters arising from a force balance is the appropriate parameter for this problem for a wide range of viscosity and electrical conductivity. The same group of parameters is used to correlate the non-dimensional frequency of droplet formation, ${N}_{d}^{\ast}$, which has a gradual slope for *Ca*_*e*_/$\sqrt{Bo(1+Ca)} < 2$, while it behaves linearly when the electric force is dominant over the other forces, i.e. when *Ca*_*e*_/$\sqrt{Bo(1+Ca)} > 3$. This change in slopes occurs due to the formation of the conical meniscus, i.e. beyond *Ca*_*e*_/$\sqrt{Bo(1+Ca)}=2$. ${N}_{d}^{\ast}$ is almost linear with reduced scatter (Fig. 6) and compares well with the expressions in equations (14) and (15).

Thus, the dimensional droplet radius and frequency of droplet formation for different suspensions obtained from experiments spread over a wide band as a function of voltage (Figs. 2 and 3). The spread in the data can be collapsed into single curves with minimal scatter by employing convenient non-dimensional parameters (Figs. 5 and 6) as it has also been achieved for analogous problems^30^. The analysis also shows that the radius is linearly decreasing with respect to this parameter in the dripping mode (Eq. (12)) and has an inverse relationship in the microdripping mode (Eq. (13)). Based on the non-dimensional plots of the experimental results in Fig. 5, we are able to determine the correction factors *δ*_1_ and *δ*_2_ for the dripping and microdripping modes respectively. The non-dimensional frequency of droplet formation also predicts the experimental results well as shown in Fig. 6 for the same values of *δ*_1_ and *δ*_2_.

## Discussion

A regime map is provided for identifying the limits of a non-dimensional parameter to operate an electrospray process in the microdripping mode that generates uniform monodisperse microdroplets. An increase in nanoparticle concentration increases the liquid viscosity, which dampens the growth of perturbation and delays the jet breakup in the microdripping mode. However, viscous effects are not seen to be significant in determining the dropsize in the dripping mode. The effect of the physical properties on the droplet size and frequency of droplet formation is more precisely described by the relative influence of all the acting forces. Dimensional analysis has yielded a parameter, *Ca*_*e*_/$\sqrt{Bo(1+Ca)}$ which accounts for all the acting forces. Results for nine suspensions with a wide range of viscosity show that the non-dimensional droplet radius can be correlated using this parameter in both dripping and microdripping modes. The same parameter also correlates the normalized frequency of droplet formation as an increasing function in the microdripping mode. Viscosity affects the shape of the cone by resisting its deformation, thus promoting a stable microdripping mode. Reduction in surface tension decreases the drop size in dripping and microdripping modes. However, the capillary size and electrical conductivity have minimal effect on the size of the ejected droplets. Based on the analysis, it is possible to design the electrospray to produce uniform monodisperse droplets by manipulating the voltage at the electrode, for any desired nanoparticle concentration of a suspension.

## Methods

Different amounts of surfactants and dispersants had to be added to stabilize the solution to avoid aggregation of nanoparticles. The amount of surfactant molar concentration and dispersant concentration for Si was 6mM and 0.4 *g*/*L* respectively; for SiC, the amounts were 8 mM and 1 *g*/*L*. The surfactant and the anionic dispersant used were sodium dodecyl sulfate (SDS) and poly-naphthalene sulfonic acid. The surfactants and dispersants changed the surface tension of the suspension as reported in Table 1. The surface tension of the mixture of DI water and the same amount of SDS surfactant without the nanoparticles was measured to be the same as that with the nanoparticles.

**Figure 7:**
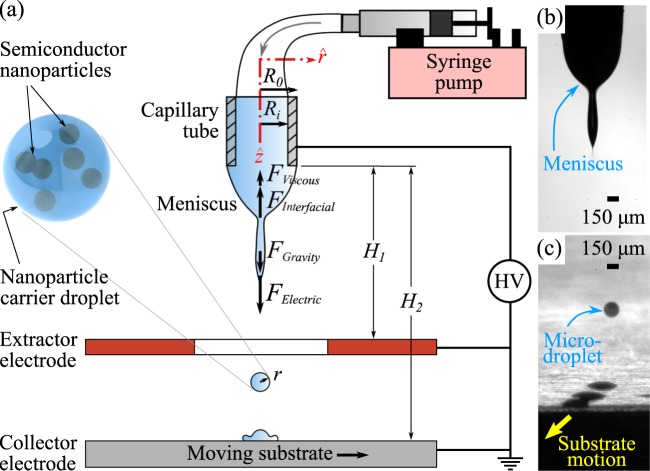
Schematic of the experimental setup. (**a**) Fundamental force balance is applied at the onset of droplet detachment. (**b**) Conical meniscus in microdripping mode. (**c**) Patterning of microdroplets using emitted drops from microdripping on a moving substrate at a constant speed.

Electrospray offers a viable method for the emission of small droplets from the detachment of liquid filaments deriving out of an elongated meniscus (Fig. 7a) caused by the action of an electric force (Fig. 7b). Patterning of the precursor suspension using the emitted droplets from the electrospray can be effected by moving the substrate at a constant speed (Fig. 7c).

The experimental setup (Fig. 7a) consists of a syringe pump connected to a capillary tube, two electrodes, and a high-voltage power supply. An electric field is applied between the stainless steel capillary tube of inner and outer radii, *R*_*i*_ = 0.255 mm and *R*_0_ = 0.41 mm respectively, and a copper disk (extractor electrode) and an aluminum substrate (collector electrode). The inner diameter, outer diameter, and thickness of the disk are 4.5 mm, 38 mm, and 0.5 mm, respectively. The tip of the capillary tube is situated at a distance *H*_1_ = 4 mm above the copper disk, and *H*_2_ = 74 mm above the substrate. The droplet size and the meniscus frequency, i.e., the formation rate of droplets, are obtained by processing the droplet images that are recorded with a high-speed camera operating at the frame rates of 2000–11000 fps and a spatial resolution of 5.5 *μ*m/pixel.

**Figure 8:**
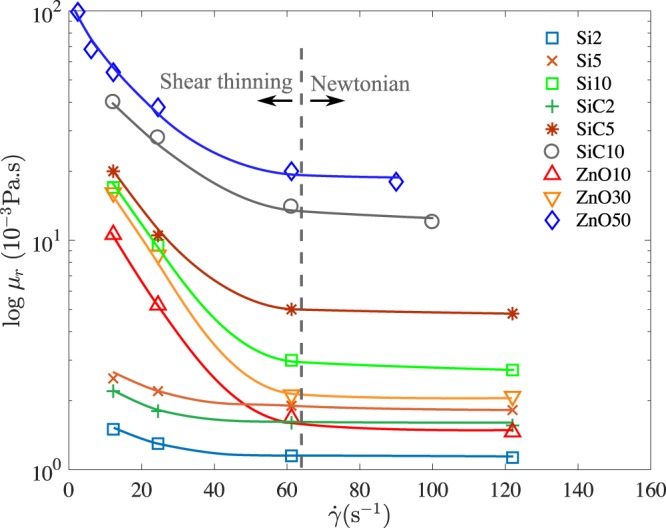
Relative viscosity of semiconductor nanosuspensions versus shear rate. The numerical suffixes denote wt% of the semiconductor materials.

All suspensions were made by dispersing the semiconductor nanoparticles [silicon (Si), silicon carbide (SiC), and zinc oxide (ZnO)] in de-ionized water with a surfactant [sodium dodecyl sulfate (SDS)] and dispersant (sodium salt of poly-naphthalene sulfonic acid) and sonicating the resulting mixture for 1 hr at an ultrasonic frequency of 20 kHz. The physical properties of these suspensions are listed in Table 1. All samples, especially those suspensions with high nanoparticle concentration (e.g., ZnO, 50 wt% in DI water), display shear thinning yield stress behavior at low shear rates. However, at relatively high shear rates, the hydrodynamic interactions between the dispersed particles and medium are predominant, leading to Newtonian behavior. The values of the shear rate for the occurrence of Newtonian flow was determined from experimental data on the relative viscosity vs. shear rate. The value at which the suspensions start to display Newtonian behavior was found to vary from 50 s^−1^ to 60 s^−1^. Using the Hagen-Poiseuille flow model within the capillary tube, $\dot{\gamma }=2Q$/$\pi {R}_{i}^{3}$, where $\dot{\gamma }$ and *Q* are the average shear rate and the volumetric flow rate of the suspension inside the capillary tube, respectively. The volumetric flow rate of 1.67 mm^3^/s was found to yield an average shear rate of 64 s^−1^, beyond which the fluid is Newtonian as seen from the experiments in Fig. 8.

Nanoparticles used in suspension were between 10 *nm* and 30 *nm* in diameter, significantly smaller than the drop size of less than 100 *μm* in the microdripping regime. Therefore, no clogging was observed for the flow rates used due to the presence of nanoparticles. The increase in particle concentration increases the viscosity of the suspension, inhibits the jet breakup and slows down the frequency of droplet ejection with increasing Ca as the model has shown. However, clogging occurred at lower volumetric flow rates at the tip of the capillary tube since the suspensions exhibit a pseudoplastic behavior manifesting their higher viscosity. In general, viscosity of the suspension was increased by increasing the nanoparticle concentration that dampens the growth of perturbation in the Taylor cone^31^.

## Appendix

### Electric force

The electric force on the droplet is estimated as, *F*_*Electric*_ = *Sε*_0_*ε*_*r*_*E*^2^/2, where *S*, *ε*_0_, *ε*_*r*_, and *E* are the surface area of the droplet (*S* = 4*πr*^2^), electric permittivity of vacuum, characteristic relative permittivity of each aqueous suspension, and the externally applied electric field^32^. The characteristic relative permittivity is defined as *ε*_*r*_ = 1 + *σ*/(*ε*_0_*ω*) based on the Lorentz model for the interaction of electromagnetic waves in dielectric materials^32,33^, where *σ* and *ω* are the electrical conductivity of each aqueous suspension and characteristic frequency taken as *c*/*L* where *c* and *L* are the speed of light in the air and the distance from the center of the capillary tip and the inner edge of the extractor electrode.

The applied electric field is estimated as *E* = *E*_1_ + *E*_2_ for the three-electrode system of this study, where *E*_1_ and *E*_2_ are the electric fields between the capillary tube and (1) the extractor electrode and (2) the collector electrode. ${E}_{1}=\sqrt{2}\phi$/*R*_0_*ln*(4*H*_1_∕*R*_0_) and ${E}_{2}=\sqrt{2}\phi$/*R*_0_*ln*(4*H*_2_∕*R*_0_), where *ϕ* is the applied electric voltage^34^.
